# Auricular Acupressure for Dry Eye Disease: A Systematic Review and Meta-Analysis of Randomized Controlled Trials

**DOI:** 10.3390/medicina59010177

**Published:** 2023-01-16

**Authors:** Qiong Huang, Mengqi Zhan, Zhe Hu

**Affiliations:** Department of Ophthalmology, Renmin Hospital of Wuhan University, Wuhan 430060, China

**Keywords:** auricular acupressure, dry eye disease, systematic review

## Abstract

*Background and Objectives*: The purpose of this systematic review was to summarize the current evidence to examine the safety and effectiveness of auricular acupressure on dry eye diseases. *Materials and Methods*: Twenty databases were searched from their inception until November 2022. Only randomized controlled trials (RCTs) in which auricular acupressure was used for dry eye diseases were included. The selection process, data extraction and quantitative were conducted according to the guidelines. *Results*: Seven RCTs met the inclusion criteria. Meta-analysis showed that compared to artificial tears, auricular acupressure had a favorable effect on prolonging tear breakup time (TBUT), improving the Schirmer I test (SIT) score and the score of symptoms (SOS) of patients with dry eye disease (*p* < 0.05). Furthermore, compared to the artificial tears alone, auricular acupressure plus artificial tears had a significantly greater SIT score (*p* < 0.001) and response rate (*p* = 0.006), significantly longer TBUT (*p* < 0.001), and significantly lower Ocular surface disease index (OSDI) (*p* = 0.02) and SOS (*p* = 0.03). However, there was no statistically significant difference between the auricular acupressure plus artificial tears group and the artificial tears group in terms of cornea fluorescein staining (CFS) (*p* = 0.09). *Conclusions*: Auricular acupressure, as a sole intervention or in combination with artificial tears, may have a beneficial effect on dry eye disease. However, more high-quality RCTs need to be included in the future to further prove the positive effects of auricular acupressure on patients with dry eye disease.

## 1. Introduction

According to the definition of TFOS DEWS II, dry eye disease is a complex and multifactorial ophthalmological condition that is characterized by low tear production, abnormal homeostasis of the tear film, tear hypertonicity, and ocular surface inflammation and damage [1]. Dry eye diseases affect approximately 5% to 35% of the adult population in the world [2], and the total prevalence rate of dry eye diseases in China was reported to be greater than 30% [3]. According to a recent survey, more than 20% of outpatients in Korean ophthalmologic clinics were diagnosed with dry eye disease [4], while more than 30% of outpatients in Chinese ophthalmologic clinics were diagnosed with dry eye disease [5]. Furthermore, in the United States of America, about 16.4 million adults have been diagnosed with dry eye disease. In addition, 6 million American adults have experienced dry eye symptoms although they have not been diagnosed with dry eye disease [6].

Dry eye disease can occur as an isolated disease or co-morbid to systemic diseases (diabetes, Sjögren’s syndrome, fibromyalgia, etc.) [7]. Common symptoms of dry eye diseases include redness and dryness of the eyes, poor visual quality, photophobia, itchy eyes, ocular irritation, fear of the wind, a foreign body sensation, eye fatigue, pain perception, lower vitality, etc. [8,9]. According to a recent survey by Sriprasert et al., women, particularly postmenopausal women, reported a higher prevalence of dry eye diseases than men [10]. Dry eye diseases not only restrict patients’ individual daily activities (reading, performing professional work, the use of electronic devices, watching television, driving, etc.) and impair the quality of life and mental health of patients, but also have a negative impact on social productivity and work efficiency, which ultimately results in a serious economic burden on patients’ family and social healthcare system [11,12,13]. For example, a current pharmacoeconomic analysis showed that the economic burden of dry eye diseases in the American healthcare system is estimated to be over $55.4 billion [14].

To date, TFOS DEWS II and the American Academy of Ophthalmology have recommended over-the-counter artificial tears (with and without lipid components) and topical 0.05% cyclosporine A (CsA) as the optional treatment modality for patients with dry eye diseases [15,16,17]. Although over-the-counter artificial tears can alleviate symptoms of dry eye diseases by reducing inflammation and tear-film hyperosmolarity, their temporary clinical efficacy can only last 30 to 40 min and need to be used frequently [18]. The USA Food and Drug Administration approved topical 0.05% CsA for the treatment of dry eye diseases in 2003. Topical 0.05% CsA has been reported to improve the scores of the ocular surface disease index (OSDI) and tear-film breakup time (TBUT) in patients with dry eye diseases by reducing IL-2 mediated T-cell activation [19]. However, up to 17% of patients with dry eye disease reported ocular burn after using topical 0.05% CsA, which prevented some patients from using topical 0.05% CsA for the management of their dry eye diseases [20].

Therefore, patients with dry eye diseases often turn to complementary and alternative medicine (CAM) for help [21]. Smith et al. [22] reported that about 42% of Australian patients with inflammatory eye disease had applied CAM. As a key component of CAM, acupuncture has been recognized as a promising treatment modality for dry eye disease patients [23]. According to the recommendations of the WHO international guideline, acupuncture can be used for a variety of eye diseases, including acute conjunctivitis, central retinitis, myopia, and cataracts [24]. Additionally, an updated systematic review has shown that compared to the over-the-counter artificial tears, acupuncture could significantly prolong tear breakup time and increase the Schirmer I test (SIT) score (*p* < 0.05) [25]. Furthermore, the results of the study by Kim et al. [26] revealed that the effects of acupuncture lasted longer than those of over-the-counter artificial tears. However, dry eye disease patients who want to receive an acupuncture intervention must go to an acupuncture clinic and spend time there, which increases the time burden of dry eye patients and the economic burden of travel. Thus, Kim et al. [26] reported that fewer patients with dry eye disease visited acupuncture clinics to manage their symptoms during the study follow-up period. Furthermore, during the COVID-19 pandemic, patients with dry eye diseases frequently returned home and acupuncture clinics will increase the risk of infection.

Auricular acupressure is also an acupuncture-related technique that stimulates acupuncture points on the ear with magnetic pellets, rather than a metal acupuncture needle [27]. Auricular acupressure has been shown to be effective in the management of ophthalmic diseases, including myopia [28,29]. Compared to acupuncture, auricular acupressure has the advantage of being non-invasive, aseptic, and easily self-administered at home. Thus, the convenience advantage of auricular acupressure exceeds that of acupuncture, especially during the COVID-19 pandemic [30]. Previously, Pesavento et al. [31] reported that auricular acupressure had a lasting effect on improving tear-film production, and balancing the tear’s aqueous and mucosal components. Therefore, it is reasonable to assume that auricular acupressure not only could maintain the same lasting effect as acupuncture, but also had convenience characteristics, especially suitable for patients with dry eye disease to be self-administrated at home during the COVID-19 pandemic period [32]. To prove the above hypothesis, therefore, the objective of this systematic review was to summarize and critically assess evidence from randomized clinical trials (RCTs) of auricular acupressure in the treatment of dry eye diseases according to the Preferred Reporting Items for Systematic Reviews and Meta-Analyses (PRISMA) statement.

## 2. Materials and Methods

This systematic review was performed following the principle of PRISMA [33] (Supplementary File S1). Additionally, the protocol of this systematic review has been registered with OSF registration (Registration Number: osf.io/gz2vn (accessed on 2 November 2022)).

### 2.1. Data Sources and Search Strategy

The following databases were searched from their inception until November 2022, without language restrictions: PubMed, EMBASE, The Cochrane Eyes and Vision Group Trials Register, Sino-Med, CiNii, KoreaMed, the Cochrane Central Register, CINAHL, and AMED.

The key search terms included: ‘dry eye syndromes’, ‘xerophthalmia’, ‘vitamin A defi ciency’, ‘keratoconjunctivitis sicca’, ‘keratoconjunctivitis’, ‘acupressure’, ‘er xue ya tie’, ‘ear-apex’, ‘ear acupuncture’, ‘electroacupuncture’, ‘randomized controlled trial’, and ‘controlled clinical trial’. Search strategies were listed in Supplementary File S2 (PubMed).

### 2.2. Study Selection

This systematic review included RCTs that assess the effectiveness and safety of auricular acupressure for patients with dry eye disease. However, in vitro experiments, retrospective studies, and survey studies were excluded. Moreover, all included RCTs should also meet the following inclusion criteria with the PICO principle.

#### 2.2.1. P (Population)

The patients were diagnosed with dry eye disease using the TFOS DEWS II diagnostic criteria [1]. Consistent with the previous study [34], in this study, we mainly included non-specific typical dry eye disease, which is caused by exposure to video terminals, enclosed air-conditioned environments and the growth of the aging population [12]. Thus, patients with dry eye disease caused by systemic diseases (Sjögren’s syndrome or diabetes mellitus) or other specific causes (small incision lenticule extraction (SMILE) surgery) were excluded from this study.

#### 2.2.2. I (Intervention)

The studies included compared auricular acupressure with artificial tears or auricular acupressure plus artificial tears with artificial tears alone or auricular acupressure with sham auricular acupressure. Auricular acupressure combined with the other CAM therapies in the intervention group, including any types of acupuncture (electroacupuncture, laser acupuncture, warming needle acupuncture), Chinese herbs, any types of moxibustion (direct moxibustion, indirect moxibustion, thunder-fire moxibustion), massage, etc., was excluded from this systematic review.

#### 2.2.3. C (comparison)

The artificial tears intervention was confirmed as a recommended therapy for patients with dry eye disease in the control group. Moreover, a sham auricular acupressure was also included in the control group. However, the other CAM therapies, including any types of acupuncture (electroacupuncture, laser acupuncture, warming needle acupuncture), Chinese herbs, any types of moxibustion (direct moxibustion, indirect moxibustion, thunder-fire moxibustion), massage, etc., in conjunction with artificial tears or sham auricular acupressure were excluded in this systematic review.

#### 2.2.4. O (Outcome)

##### Primary Outcome

(1) Tear-film breakup time (TBUT): After applying sodium fluorescein to both eyes, the researcher measured the interval between the blink of the eyes and the first appearance of a dry spot in the tear-film. The shorter TBUT time means the worse the tear-film stability [35].

(2) Schirmer I test (SIT): The researcher placed the SIT strip on the participants’ lateral third of the lower eyelids, and asked them to close their eyes. Five min later, the researcher measured and recorded the length of tear wetting in the SIT strip. The shorter the length of tear wetting in the SIT strip means the less tear secretion from participants [36].

(3) Ocular surface disease index (OSDI): OSDI was used to examine general ocular-related symptoms for patients with dry eye disease. The OSDI included three subscales and the total OSDI score ranged between 0 and 100 points. A higher OSDI score means more serious symptoms of dry eye disease [37].

##### Secondary Outcome

(1) Cornea Fluorescein Staining (CFS): The cornea was divided into four quadrants, and the staining score of each quadrant ranged from 0 to 3 points, so the staining score of the entire cornea ranged from 0 to 12 point. The higher the CFS score means the more serious symptoms of dry eye disease [38].

(2) The score of symptoms (SOS) (DEWS 2007): SOS included 5 dimensions and 12 questions, and the total score ranged from 0 to 45 score. A higher score means more severity of dry eye symptoms [39].

(3) Response rate: The TCM efficacy assessment tool, guiding principles of clinical research on new drugs (GPCRND), was used to assess the response rate of auricular acupressure in patients with dry eye disease in this study [40].

(4) Adverse Events (AEs).

### 2.3. Screening Procedures of Eligible Studies and Data Extraction

#### 2.3.1. Screening Procedures of Eligible Studies

Two review authors (QH and MQZ) independently assessed abstracts and titles of studies identified by the literature search. Duplicates were omitted using Noteexpress software. Relevant studies were selected against the predefined inclusion criteria. If necessary, reviewers would examine full-text reports to identify eligible studies. Noteexpress software was also used to manage records. Any disagreement was confirmed by the third reviewer (ZH).

#### 2.3.2. Data Extraction

Data extraction was performed with a pre-piloted, standardized form by two independent reviewers (QH and MQZ). For each trial, the specific extracted information included study designs, author names, publication years and the country, sample size, Gender, intervention regimens, control regimens, acupuncture points selection, main outcomes, and adverse events. Any disagreement was confirmed by the third reviewer (ZH).

### 2.4. Assessment of Risk of Bias in Included Studies

Two authors (QH and MQZ) independently assessed the risk of bias using the Cochrane Handbook criteria. The following risk of bias domains were assessed: sequence generation (selection bias); allocation concealment (selection bias); blinding of participants and personnel (performance bias); blinding of outcome assessment (detection bias); incomplete outcome data (attrition bias); selective outcome reporting (reporting bias); and other bias. The final decisions were made by a third reviewer (ZH) if inconsistent results appeared.

### 2.5. Quality of Evidence

Two independent reviewers applied the Grades of Recommendations, Assessment, Development, and Evaluation (GRADE) framework recommended by the Cochrane Collaborative Organization to comprehensively appraise the evidence quality of each outcome index [41,42,43]. Evidence was downgraded for the following aspects: (1) high risk of bias (RoB) of included studies, (2) inconsistency of results, (3) indirectness, (4) imprecision, and (5) publication bias. Based on the above judgments, the quality of the evidence for each outcome was classified as ‘high’, ‘moderate’, ‘low’, or ‘very low’ according to the number of downgraded levels (initial grading for ‘high’, the downgrade by one level for ‘moderate’, the downgrade by two levels for ‘low’, and downgrade by three levels for ‘very low’). The third reviewer (ZH) acts as an arbitrator to settle any discrepancies between two independent reviewers (QH and MQZ).

### 2.6. Assessment of Heterogeneity and Data Synthesis

The *I*^2^ statistic was used to assess the heterogeneity of the included studies [44], as the criterion, *I*^2^ *<* 50%, indicates low heterogeneity, while *I*^2^ > 50% indicates high heterogeneity. The meta-analysis of intervention and outcome measure methods was conducted by RevMan software [45]. Pooled dichotomous data (response rate) would be shown as RR. Additionally, we would express the results of TBUT, SIT, SOS, CFS, and OSDI as mean differences with 95% confidence intervals. If the statistical heterogeneity were low (*I*^2^ < 50%), the fixed-effect model would be used to combine the data, while if the statistical heterogeneity were high (*I*^2^ > 50%), the random-effect model would be used.

### 2.7. Sensitivity Analysis

Sensitivity analyses were conducted to explore the robustness of the meta-analysis results by varying the analytic data or methods.

### 2.8. Assessment of Reporting Bias

Publication bias would be tested if the number of included trials was greater than 10 [46].

### 2.9. Ethic Approval

No ethical issues were needed because the patients were not involved.

## 3. Results

### 3.1. Search Results and Study Characteristics

**Figure 1:**
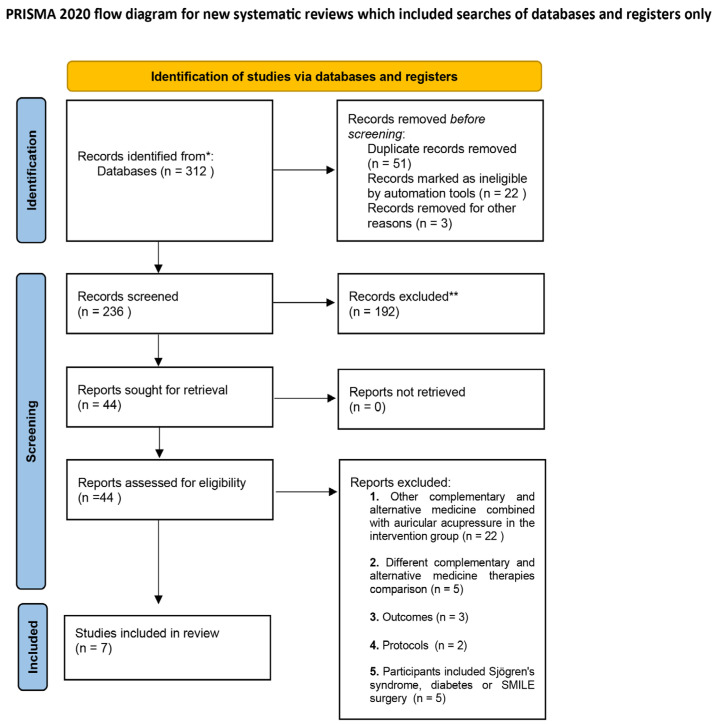
Flow diagram of the selection steps of the identified articles according to the PRISMA. * databases, ** how many records excluded.

When databases were initially searched, 312 relevant trials were obtained. After removing duplicated published trials and ineligible trials, a total of 236 trials were further screened and reviewed. After a further screening of the titles and abstracts of 236 trials, 192 trials were excluded. We conducted a full-text search and review of the remaining 44 trials and found that 22 trials used other complementary and alternative medicine therapies (Chinese herbs, acupuncture, moxibustion, *Guasha*, etc.) in conjunction with auricular acupressure in the intervention group, five trials compared different CAM therapies for dry eye diseases, and the outcomes of three trials did not conform to the inclusion criteria of this study, two trials were research protocols, and five trials included patients with dry eye diseases caused by Sjogren’s syndrome, diabetes, and SMILE surgery. After deleting the 37 trials, we finally included seven trials [47,48,49,50,51,52] for the subsequent systematic review (Figure 1).

**Figure 2:**
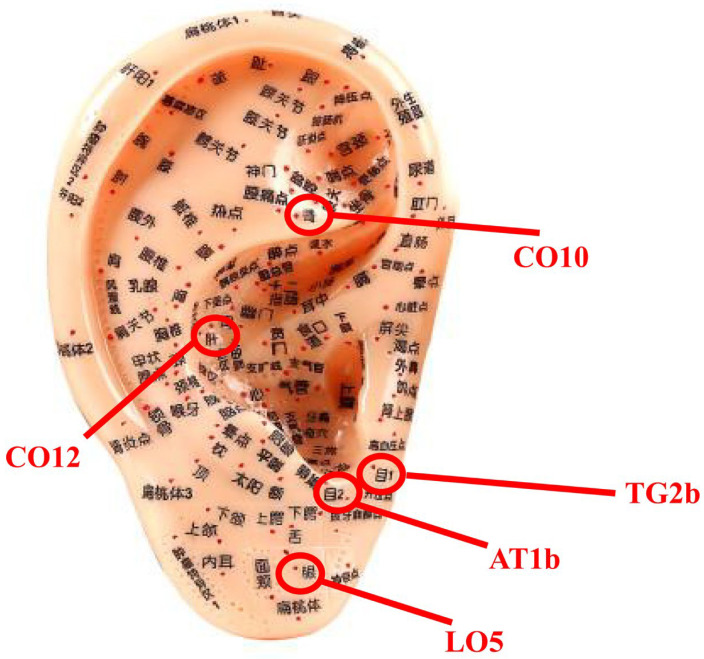
Most common acupuncture points landmarks for dry eye disease.

With the exception of one RCT [32] from the Republic of Korea, the remaining RCTs originated in China [47,48,49,50,51,52]. All RCTs have been published in the last 5 years. The sample size included in the RCT ranged from 46 to 141, and female patients significantly more than male patients. Three RCTs tested auricular acupressure alone [47,48,49]; auricular acupressure combined with artificial tears was used in three RCTs [50,51,52]; only one RCT compared the effects of auricular acupressure and sham auricular acupressure on patients with dry eye disease [32]. One RCT [48] used polyethylene glycol eye drops for artificial tears, two RCTs [51,52] applied sodium hyaluronate eye drops for artificial tears, and the other RCTs did not mention the type of artificial eye drops used. The duration of the interventions ranged from 2 weeks to 8 weeks. The selection of acupuncture points for auricular acupressure was based on the theory of TCM, and the acupuncture points most used for auricular acupressure to alleviate dry eye disease were *Mu 1* (TG2b), *Mu 2* (AT1b), Kidney (CO10), and Liver (CO12) (Figure 2). The details of the included RCTs are summarized in Table 1.

### 3.2. Risk of Bias and Quality of Evidence

**Figure 3:**
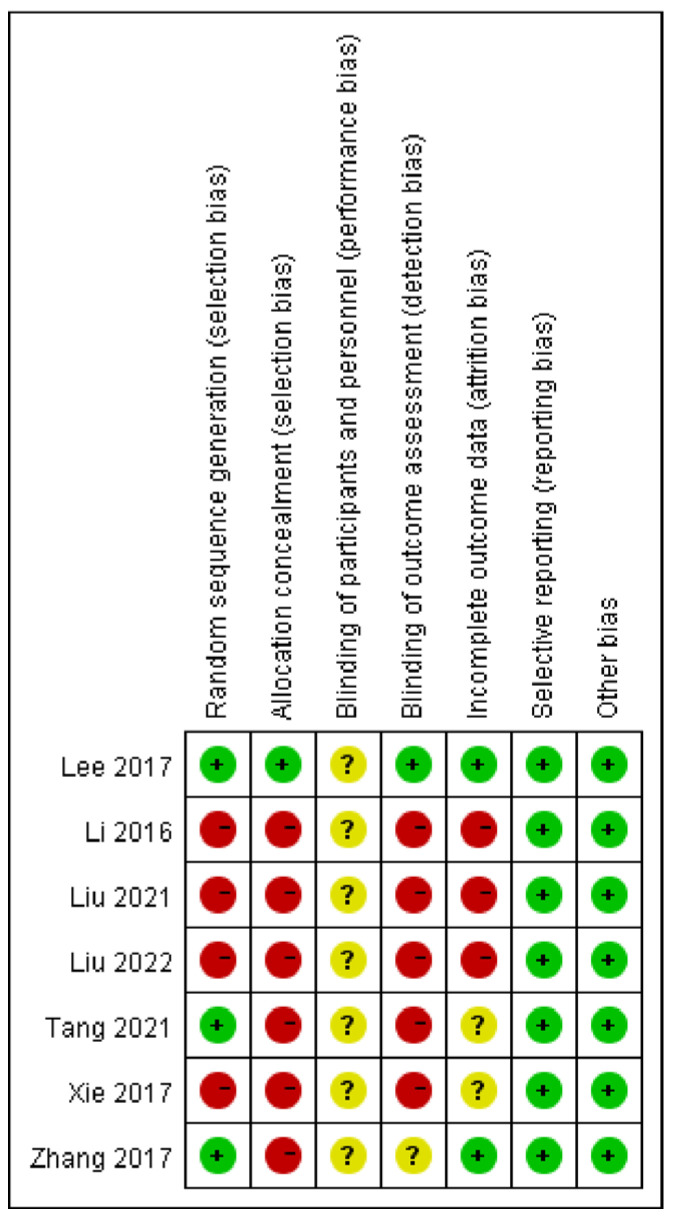
Risk of bias graph: review authors’ judgments about each risk of bias item presented as percentages across all included studies. Green: Low risk of bias; Yellow: Unclear risk of bias; Red: High risk of bias [32,47,48,49,50,51,52].

**Figure 4:**
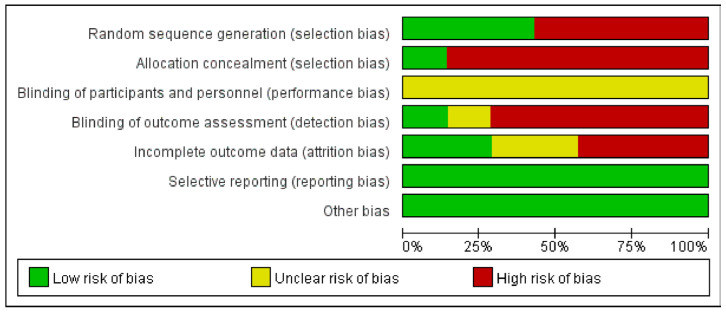
Risk of bias summary: review authors’ judgments about each risk of bias item for each included study.

The Cochrane risk of bias is presented in Figure 3 and Figure 4. Only three RCTs [32,51,52] reported appropriate random sequence generation, three RCTs [48,49,50] only mentioned ‘randomization’, but no specific ‘random sequence generation’ method was reported, while the remaining one RCT [47] did not describe the methods of random sequence generation. Only one RCT [32] carried out the concealment of allocation by sealed envelopes. Due to the nature of auricular acupressure interventions, it is not possible to blind participants and performers. However, only one RCT [32] reported the blinding of the assessor and two RCTs [32,52] reported the withdrawal of the participant. Selective reporting results were reported in all RCTs.

The quality of evidence for TBUT, SIT, and CFS ranged from low to very low. Risk of bias and imprecision were the most common downgrading factor, followed by inconsistency, indirectness, and publication bias (Table 2).

### 3.3. Quantitative Data Synthesis

#### 3.3.1. Artificial Tears vs. Auricular Acupressure

##### TBUT

**Figure 5:**
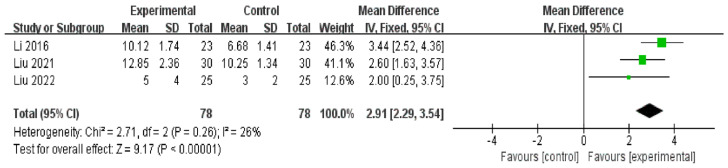
Artificial tears vs. Auricular acupressure on TBUT [47,48,49].

There were three RCTs [47,48,49] (*n* = 156) in which TUBT was used as a measurement of the effects of auricular acupressure on dry eye disease patients. Meta-analysis suggested that compared to artificial tears, auricular acupressure had a favorable effect on improving tear-film stability of dry eye disease patients [MD = 2.91, 95% CI (2.29 3.54), *p* < 0.001, with low heterogeneity: Chi^2^ = 2.71, *p* = 0.26, *I*^2^ = 26%] (Figure 5).

##### SIT

**Figure 6:**
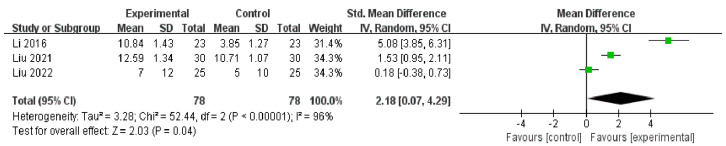
Artificial tears vs. Auricular acupressure on SIT [47,48,49].

There were three RCTs [47,48,49] (*n* = 156) that used SIT as the outcome to explore the effect of auricular acupressure on patients with dry eye diseases. Two of the RCTs [47,48] indicated positive effects on SIT between groups, while the remaining one [49] did not. The meta-analysis suggested that, compared to the artificial tears intervention, auricular acupressure had a favorable effect on SIT [MD = 2.18, 95% CI (0.07, 4.29), *p* = 0.04], with high heterogeneity [Chi^2^ = 52.44, *p* < 0.01, *I*^2^ = 96%] (Figure 6).

##### SOS

An RCT [48] (*n* = 46) used SOS as a measure to test the effect of auricular acupressure on the severity of symptoms in dry eye disease patients. Compared to artificial tears, auricular acupressure had positive effects in reducing the severity of symptoms in patients with dry eye diseases [MD = −11.5, 95% CI (−13.68, −9.32), *p* < 0.01].

##### OSDI

An RCT [49] (*n* = 50) used OSDI as a measure to examine the effect of auricular acupressure on general ocular-related symptoms for dry eye disease patients. Compared to artificial tears, auricular acupressure had no significant effects in improving OSDI for patients with dry eye diseases [MD = −8.22, 95% CI (−16.90, 0.46), *p* = 0.06].

#### 3.3.2. Artificial Tears vs. Auricular Acupressure plus Artificial Tears

##### TBUT

**Figure 7:**
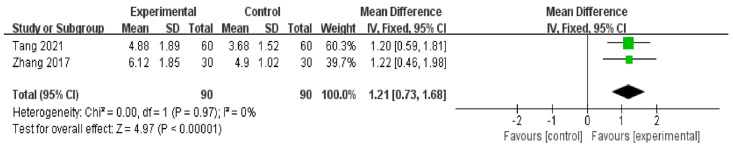
Artificial tears vs. Artificial tears plus Auricular acupressure on TBUT [51,52].

There were two RCTs [51,52] (*n* = 180) in which TUBT was used as a measurement of the effects of auricular acupressure on dry eye disease patients. Meta-analysis suggested that compared to artificial tears alone, auricular acupressure plus artificial tears had a favorable effect on improving tear-film stability in patients with dry eye disease. [MD = 1.21, 95% CI (0.73, 1.68), *p* < 0.001, with low heterogeneity: Chi^2^ = 0.00, *p* = 0.97, *I*^2^ = 0%] (Figure 7).

##### SIT

**Figure 8:**
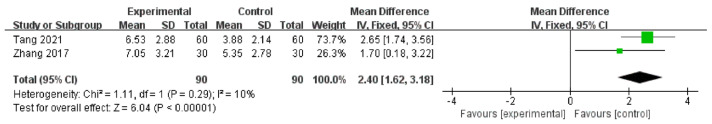
Artificial tears vs. Artificial tears plus Auricular acupressure on SIT [51,52].

There were two RCTs [51,52] (*n* = 180) in which SIT was used as a measurement of the effects of auricular acupressure on dry eye disease patients. The meta-analysis suggested that, compared to artificial tears alone, auricular acupressure plus artificial tears had a favorable effect on increasing the quantity of tear secretion of dry eye disease patients. [MD = 2.40, 95% CI (1.62, 3.18), *p* < 0.001, with low heterogeneity: Chi^2^ = 1.11, *p* = 0.29, *I*^2^ = 10%] (Figure 8).

##### CFS

**Figure 9:**
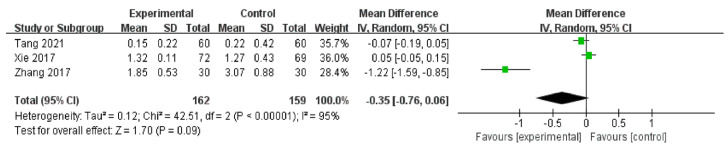
Artificial tears vs. Artificial tears plus Auricular acupressure on CFS [50,51,52].

There were three RCTs [50,51,52] (*n* = 321) that used CFS as the outcome to explore the effect of auricular acupressure on patients with dry eye diseases. Two of the RCTs [50,51] indicated positive effects on CFS between groups, while the remaining one [52] did not. The meta-analysis suggested that compared to artificial tears intervention alone, auricular acupressure plus artificial tears had no statistically significant effect on CFS [MD = −0.35, 95% CI (−0.76, 0.06), *p* = 0.09], with high heterogeneity [Chi^2^ = 42.51, *p* < 0.01, *I*^2^ = 95%] (Figure 9).

##### Response Rate

Only one RCT [50] (*n* = 141) used the response rate as a measure to test the effect of auricular acupressure on treatment efficacy in dry eye disease patients. Compared to artificial tears, auricular acupressure plus artificial tears had positive effects in improving the treatment efficacy for patients with dry eye diseases [RR = 1.33, 95% CI (1.08, 1.63), *p* = 0.006].

##### SOS

Only one RCT [52] (*n* = 60) used SOS as a measure to test the effect of auricular acupressure on the severity of symptoms in patients with dry eye diseases. Compared to artificial tears, auricular acupressure plus artificial tears had positive effects in reducing the severity of symptoms in patients with dry eye diseases [MD = −3.11, 95% CI (−5.97, −0.25), *p* = 0.03].

##### OSDI

Only one RCT [52] (*n* = 60) used OSDI as a measure to examine the effect of auricular acupressure on general ocular-related symptoms for dry eye disease patients. Compared to artificial tears, auricular acupressure plus artificial tears had significant effects in improving OSDI for patients with dry eye diseases [MD = −4.95, 95% CI (−8.94, −0.96), *p* = 0.02].

#### 3.3.3. Auricular Acupressure vs. Sham Auricular Acupressure

Only one RCT [32] compared the effects of auricular acupressure and sham auricular acupressure on patients with dry eye disease. The results suggested that, compared to sham auricular acupressure, auricular acupressure had a favorable effect on improving OSDI [MD = −0.84, 95% CI (−1.32, −0.36), *p* < 0.01], TBUT [MD = 2.61, 95% CI (1.74, 3.48), *p* < 0.01], and SIT [MD = 1.63, 95% CI (0.85, 2.41), *p* < 0.01] of patients with dry eye diseases.

### 3.4. Sensitive Analysis

To explore possible heterogeneity, we performed a sensitivity analysis for the outcome of SIT in the artificial tears versus auricular acupressure section (*I*^2^ = 96%) and CFS in the artificial tears versus auricular acupressure plus artificial tears section (*I*^2^ = 95%). We tried to carry out the sensitivity analysis based on the different intervention times (Less than 4 weeks vs. over 4 weeks). The results suggested that, in the artificial tears vs. auricular acupressure section, the heterogeneity of the SIT index decreased from *I*^2^ = 96% to *I*^2^ = 0%. However, in the artificial tears vs. auricular acupressure plus artificial tears section, the heterogeneity of CFS outcomes has not decreased (*I*^2^ = 98%) (Table 3).

### 3.5. Adverse Events

Only one RCT [32] reported that participants in the auricular acupressure group experienced mild, pricking pain on the local skin. However, the remaining RCTs [47,48,49,50,51,52] did not evaluate the safety of auricular acupressure for dry eye disease patients.

## 4. Discussion

### 4.1. Principal Findings

The main findings of this systematic review suggested that compared to artificial tears, auricular acupressure had a favorable effect on prolonging the TBUT and improving the SIT score of dry eye disease patients (*p* < 0.05). SOS was significantly lower in the auricular acupressure group than in the artificial tears group (*p* < 0.05). However, there was no statistically significant difference between the auricular acupressure group and the artificial tears group in terms of OSDI (*p* > 0.05). Moreover, compared to the artificial tears alone, auricular acupressure plus artificial tears had significantly greater SIT score (*p* < 0.001) and response rate (*p =* 0.006), significantly longer TBUT (*p* < 0.001), and significantly lower OSDI (*p* = 0.02) and SOS (*p* = 0.03). However, there was no statistically significant difference between the auricular acupressure plus artificial tears group and the artificial tears group in terms of CFS (*p* = 0.09). Due to the small number and the overall low quality of the RCTs included in this systematic review, these findings should be concluded with caution.

### 4.2. Comparison with the Previous Literature

As far as we are concerned, there is no systematic review evaluating the effectiveness of auricular acupressure in treating dry eye diseases. Previously, there have been several meta-analysis studies that examine the effectiveness of acupuncture on dry eye diseases. In agreement with the results of our systematic review, the results of the previous three meta-analysis studies [23,34,53] showed significant increases in the SIT score and significant decreases in OSDI (*p* < 0.05). However, only one systematic review [34] reported that acupuncture in combination with artificial tears significantly prolonged TBUT compared to artificial tears alone, which was inconsistent with the results of our systematic review (*p* < 0.05). In general, acupuncture combined with artificial tears has a strong synergistic effect on tear secretion but a weak effect on tear-film stability. However, unlike acupuncture, auricular acupressure plus artificial tears had a strong synergetic effect on both tear secretion and tear-film stability (Figure 7 and Figure 8).

### 4.3. Risk of Bias and Evidence Quality

Overall, all included RCTs had a high risk of bias, which suggested an absence of a high level of evidence. For allocation concealment, the group assignment was adequately concealed in only one included RCT [32], which can overestimate the results that should be [54]. It is not possible to blind the performers due to the nature of auricular acupressure interventions. However, only one included RCT [32] mentioned blinding assessors. For attrition bias, only 28.57% of the included RCTs reported an incomplete outcome, which could result in attrition bias [55]. Ethics is another issue that needs to be focused on. In our systematic review, only one RCT [32] mentioned that this clinical trial was approved by the Investigational Review Board of the local university hospital. However, other RCTs from China did not mention ethical considerations. Therefore, CAM practitioners from China need to strengthen their training to enhance the awareness of patient rights protection and understanding of ethical issues.

### 4.4. Exploration of Heterogeneity

As in the previous study on acupuncture [53], the heterogeneity in the effects of auricular acupressure on CFS (*I*^2^ = 96%) of dry eye disease was high. To reduce the possible heterogeneity of the current results, our systematic review has set strict inclusion and exclusion criteria. For example, auricular acupressure combined with other CAM interventions, including any types of acupuncture (electroacupuncture, laser acupuncture, warming needle acupuncture), Chinese herbs, any types of moxibustion (direct moxibustion, indirect moxibustion, thunder-fire moxibustion), massage, etc., was excluded from this systematic review. Moreover, patients with Sjögren’s syndrome or diabetes mellitus suffering from dry eye syndromes were also excluded from this systematic review. Furthermore, we also conducted a subgroup analysis and a sensitivity analysis for different study designs (auricular acupressure vs. artificial tears or auricular acupressure plus artificial tears vs. artificial tears alone) and different treatment durations (less than 4 weeks vs. over 4 weeks) (Table 1 and Table 2). Although our research team has made a lot of efforts, the problem of high heterogeneity in the outcome of CFS (*I*^2^ = 96%) was not completely resolved. To explore this issue, different auricular acupressure practitioners, different acupuncture points selection, unreported allocation concealment, different durations of dry eye syndrome, different follow-up times, and different artificial tears usage are important contributors to the high heterogeneity and affect the evaluation of the present results.

### 4.5. The Cumulative and Lasting Effects of Auricular Acupressure

The cumulative and lasting effects of acupuncture-related technologies on the improvement of symptoms and parameters of dry eye disease have been proved by several RCTs [26,56]. Kim et al. [26] and Gong et al. [56] reported that, compared to the artificial tears control group, the acupuncture group had no significant differences in SIT, and TBUT after immediate treatments, but the above indicators showed significant improvement after acupuncture follow-up treatment, suggesting the delayed effect of acupuncture treatment. In our review, two RCTs of Lee et al. [32] and Li et al. [48] evaluated the immediate and cumulative and lasting effect of the auricular acupressure intervention, and the results of this study are somewhat in line with those of previous acupuncture related clinical trials [26,56]. Lee et al. [32] reported that there were no significant differences between the auricular acupressure group and the artificial tears group after treatment in terms of SIT scores, but at the 12-week follow-up, improvements were observed in the auricular acupressure group. Li et al. [48] reported that there were no significant differences between the auricular acupressure group and the artificial tears group after 2 weeks of treatment in terms of TBUT, but at the 8 weeks of follow-up, improvements in the TBUT parameters were observed in the auricular acupressure group. To explore this issue, the sense of *Deqi* might be the key to achieving cumulative and lasting effects after acupuncture-related techniques [57]. Patients who experience acupuncture point stimulation often report multidimensional feelings, such as dull pain, warmth, numbness, and pressure. According to recordings from the classic TCM literature of *Huang di nei jing*, the cumulative and lasting effects of acupuncture-related techniques depend on the arrival of *Qi*. The fMRI studies also showed that the effect of acupuncture-related techniques with *Deqi* still affected brain activity for a period of time even after the stop of the intervention of acupuncture-related techniques [58].

### 4.6. Safety Assessment and Placebo Effect

Although auricular acupressure is a relatively safe procedure for the management of dry eye diseases, some solitary cases of local skin itch and irritation, pain, and dizziness have been reported in a previous systematic review [59]. In our research, only one RCT [32] reported that two participants in the auricular acupressure group experienced mild pain at the auricular acupuncture points, which was somewhat in line with the previous clinical trial [60]. However, the remaining RCTs in this systematic review did not report adverse events [47,48,49,50,51,52]. In the future, we strongly recommend that all clinical trials evaluate more details related to adverse events associated with auricular acupressure.

Placebo auricular acupressure could identify the specific effects of real auricular acupressure and sham auricular acupressure for the management of dry eye diseases. However, only one RCT [32] set sham AA in the control group, the remaining RCTs did not set the placebo control group. Nevertheless, even acupressure at non-acupuncture points can also lead to physiologic change beyond that of a placebo effect and activate some sensory pathways [61]. Therefore, in the future, adequate placebo auricular acupressure methods are still warranted.

### 4.7. Possible Mechanism of Action of Auricular Acupressure

From the perspective of TCM, dry eye is classified as ‘*Baisezheng*’ and ‘*Shenshuijiangku*’ in ancient medical books. In the ancient medical book ‘*Shenshiyaohan*’, ‘*Baisezheng*’ is described as ‘the *Baijing* eyes are often dry and uncomfortable, without redness or swelling, blink frequently, slightly photophobic, burning and itching’. In the ancient medical book ‘*Yankedaquan*’, ‘*Shenshuijiangku*’ is described as the evil fire is steaming and burning, causing loss of *Qi* and *Jingye*, and *Jing Qi* cannot be injected into *Kongqiao* (including eyes). According to the TCM theory, dry eye diseases are attributed to the deficiency of yin in the liver and kidney, which eventually leads to systematic deficiency of yin and fluid, and the loss of nutrition in the eyes. In this systematic review, the most used acupuncture points of auricular acupressure to alleviate dry eye disease were *Mu 1* (TG2b), *Mu 2* (AT1b), Kidney (CO10), and Liver (CO12). *Mu 1* (TG2b) and *Mu 2* (AT1b) correspond to the local acupuncture points of the eye. Auricular acupressure for these acupuncture points regulates the *Qi* and blood around the eye and improves eye symptoms. Furthermore, auricular acupressure at Kidney (CO10), and Liver (CO12) can nourish the liver and kidney, and harmonize *Qi* and blood of the eyes.

In modern research, acupuncture has been shown to alleviate pro-inflammatory cytokines (interleukin (IL)-1, interleukin (IL)-6, tumor necrosis factor (TNF), and other inflammatory cytokines), thereby exerting anti-inflammatory effects and improving symptoms of dry eye diseases [62]. Moreover, the mechanisms underlying auricular acupressure in dry eye diseases are associated with increasing the quantity of tear protein, regulating hormone levels, reducing pain intensity, enhancing the level of neuropeptides, altering the level of acetylcholine in the lacrimal gland, and improving the microcirculation of the ocular surface [56,63,64]. In a recent dry-eye rabbit model study, both acupuncture and electronic acupuncture had a positive effect on inhibiting the apoptosis of lacrimal epithelial cells by decreasing the levels of transforming growth factor beta (TGF-β) [65,66]. Furthermore, using the high-throughput microarray technique, Zhang et al. [67] identified four potential target proteins (MCP-1, M-CSF, RANTES, and TIMP-1) that might be involved in the immunological mechanism of acupuncture-related techniques for dry eye diseases. Although a number of studies have preliminarily explored the modern biological mechanism of acupuncture-related technologies on dry eye diseases. However, there is still a great distance to go before we fully understand the mechanism involved in auricular acupressure for dry eye diseases.

### 4.8. Limitations

This study had several limitations that should not be ignored. Firstly, this systematic review had a high risk of bias. In the future, auricular acupressure required more Level 1 RCTs to be widely accepted as a potential intervention modality for dry eye disease [34,68]. Secondly, symptom duration, follow-up period, treatment session, and treatment frequency of auricular acupressure in each included RCT were not perfectly matched, which means that these factors leading to clinical heterogeneity can ultimately affect the results of the RCTs. More clinical trials are warranted to establish an optimized intervention protocol for auricular acupressure for dry eye diseases [53]. Thirdly, in this systematic review, the proportion of women suffering from dry eye is significantly higher than that of men (Table 1), which was attributed to differences in hormonal levels between men and women [69]. However, there were no clinical trials focusing on this field. Future research should further clarify the effect of auricular acupressure on the mechanism of action of hormonal levels in patients with dry eye disease. Fourthly, although we conducted a comprehensive search to ensure that all RCTs were included, this study only included RCTs from the Republic of Korea and China, and the lack of RCTs from other countries could lead to some bias. The number of RCTs included in this systematic review is less than 10, so we cannot test publication bias using the funnel plot, but publication bias may still exist in this systematic review.

## 5. Conclusions

In conclusion, auricular acupressure can effectively prolong TBUT, improve SIT score, and improve SOS for dry eye disease patients compared to artificial tears alone. Furthermore, auricular acupressure in conjunction with artificial tears was more effective in improving TBUT, SIT, OSDI, and SOS than artificial tears alone. However, more high-quality RCTs need to be included in the future to further prove the positive effects of auricular acupressure on patients with dry eye disease.

## Figures and Tables

**Table 1:** Main characteristics of included studies.

Study Design	Study (Author, Year, and Country)	Sample Size/ Gender (F:M)	Intervention Group	Control Group	Acupuncture Points	Main Outcomes	AEs
AA vs. AT	Liu 2021 China [47]	60 (50:10)	(A) AA (AA for 1 min/time, several times/day, total 2 weeks, *n* = 30)	(B) AT (one drop or two drops per time, total 2 weeks, *n =* 30)	Sympathy (AH6a), Kidney (CO10), Liver (CO12), *Mu* 1 (TG2b), *Mu* 2 (AT1b)	1. Tear-film Breakup Time (TBUT) 2. Schirmer I test (SIT)	n.r.
	Li 2016 China [48]	46 (46:0)	(A) AA (AA time of each acupuncture point should not be less than 30 s, AA for *Deqi*/time, total 8 weeks, *n =* 23)	(B) AT (Polyethylene Glycol Eye Drops: one drop or two drops/time, 4 times/day, total 8 weeks, *n =* 23)	Kidney (CO10), Liver (CO12), Internal genitalia (TF2), Endocrine (CO18), *Pizhixia* (AT4), *Shenmen* (TF4), Eye (LO5), *Mu* 1 (TG2b), *Mu* 2 (AT1b)	1. Tear-film Breakup Time (TBUT) 2. Schirmer I test (SIT) 3. The score of symptoms (SOS)	n.r.
	Liu 2022 China [49]	50 (30:20)	(A) AA (AA for 1~2 min/time, several times/day for *Deqi*, total 4 weeks, *n* = 25)	(B) AT (one drop or two drops per time, total 4 weeks, *n* = 25)	Eye (LO5), *Mu* 1 (TG2b), *Mu* 2 (AT1b), Kindy (CO10), Liver (CO12), Spleen (CO12), Stomach (CO4), gallbladder (CO11), Heart (CO15)	1. Ocular Surface Disease Index (OSDI) 2. Tear-film Breakup Time (TBUT) 3. Schirmer I test (SIT)	n.r.
SAA plus AT vs. AT	Xie 2017 China [50]	141 (120:21)	(A) AA (AA for 1~2 min/time, several times/day for *Deqi*, total 4 weeks, n = 72). Plus (B).	(B) AT (one drop or two drops per time, total 4 weeks, *n =* 69)	Eye (LO5), *Mu* 1 (TG2b), *Mu* 2 (AT1b), Kidney (CO10), Liver (CO12), Spleen (CO12), Stomach (CO4), gallbladder (CO11), Heart (CO15)	1.Response rate 2. Cornea Fluorescein Staining (CFS)	n.r.
	Tang 2021 China [51]	120 (66:54)	(A) AA (AA for 1~2 min/time, several times/day for *Deqi*, total 8 weeks, *n* = 60). Plus (B).	(B) AT (0.1% Sodium Hyaluronate Eye Drops: one drop or two drops/time, 4 times/day, total 8 weeks, *n =* 60)	Eye (LO5), *Mu* 1 (TG2b), *Mu* 2 (AT1b), Kidney (CO10), Liver (CO12), Spleen (CO12), Stomach (CO4), Endocrine (CO18), *Pizhixia* (AT4)	1. Tear-film Breakup Time (TBUT) 2. Schirmer I test (SIT) 3. Cornea Fluorescein Staining (CFS)	n.r.
	Zhang 2017 China [52]	60 (60:0)	(A) AA (AA for 1~2 min/time, 4 times/day, total 2 weeks, *n* = 30). Plus (B).	(B) AT (0.1% Sodium Hyaluronate Eye Drops: one drop or two drops/time, 4 times/day, total 2 weeks, *n* = 30)	*Mu* 1 (TG2b), *Mu* 2 (AT1b), Kidney (CO10), Liver (CO12), Spleen (CO12)	1. Tear-film Breakup Time (TBUT) 2. Schirmer I test (SIT) 3. Cornea Fluorescein Staining (CFS) 4.Ocular Surface Disease Index (OSDI) 5. The score of symptoms (SOS)	
AA vs. Sham AA	Lee 2017 Republic of Korea [32]	100 (78:22)	(A) AA (continuous low-frequency electronic stimulation performed twice a week for 4 weeks for 30 s at each acupuncture point, *n* = 50). Plus (B).	(B) sham AA (continuous low-frequency electronic stimulation performed twice a week for 4 weeks for 30 s at each non-acupuncture point, *n =* 50).	*Shenmen* (TF4), Zero (H1), Liver (CO12), Eye (LO5)	1. Ocular Surface Disease Index (OSDI) 2. Tear-film Breakup Time (TBUT) 3. Schirmer I test (SIT)	Only 2 participants in the intervention group experienced mild, pricking pain at the auricular acupuncture points.

Note: AA: auricular acupressure, AT: artificial tears, AEs: Adverse events, F: female, M: male, n.r.: not reported.

**Table 2:** Quality of evidence in included systematic reviews with GRADE.

Study Design	Outcomes	Included RCTs/ Participants	Risk of Bias	Inconsistency	Indirectness	Imprecision	Publication Bias	Quality of Evidence
AA vs. AT	1. TBUT	3 RCTs/156 participants	Serious ^a^	Not serious	Not serious	Serious ^c^	undetected	Low
	2. SIT	3 RCTs/156 participants	Serious ^a^	Serious ^b^	Not serious	Serious ^c^	undetected	Very low
AA vs. AA + AT	1. TBUT	2 RCTs/180 participants	Serious ^a^	Not serious	Not serious	Serious ^c^	undetected	Low
	2. SIT	2 RCTs/180 participants	Serious ^a^	Not serious	Not serious	Serious ^c^	undetected	Low
	3. CFS	3 RCTs/321 participants	Serious ^a^	Serious ^b^	Not serious	Serious ^c^	undetected	Very low

Note: ^a^: The design of the experiment with a large bias in random, distributive hiding or blind; ^b^: The confidence interval overlaps less, the heterogeneity test *p* is very small, and the *I*^2^ is larger; ^c^: Small sample size (less than 400) are included.

**Table 3:** Sensitive analysis.

Variables	Number of Studies	*Overall Effects*			Heterogeneity
		MD	95% CI	*p* Value	
AT vs. AA					
SIT *Treatment sessions*					*I*^2^ = 0
Less than 4 weeks	2	1.88	[1.27, 2.46]	<0.001	
Over 4 weeks	1	6.99	[6.21, 7.77]	<0.001	
AT vs. AT + AA					
CSF *Treatment sessions*					*I*^2^ = 98
Less than 4 weeks	2	−0.57	[−1.82, 0.67]	0.37	
Over 4 weeks	1	−0.07	[−0.19, 0.05]	0.25	

Note: SIT: Schirmer I test; CFS: Cornea Fluorescein Staining.

## Data Availability

The data has been included in figures.

## Supplementary Materials

The following supporting information can be downloaded at: https://www.mdpi.com/article/10.3390/medicina59010177/s1, Supplementary File S1: PRISMA_2020_checklist; Supplementary File S2: Searching strategy. Reference [33] has been cited in Supplementary Materials.
