# Supplementary material for: Auricular Acupressure for Dry Eye Disease: A Systematic Review and Meta-Analysis of Randomized Controlled Trials
Source: Medicina (Kaunas). 2023 Jan 16;59(1):177. doi: 10.3390/medicina59010177 (PMC9865136; doi:10.3390/medicina59010177)
Supplement: Supplementary file 1 [file medicina-59-00177-s001.zip › Supplementary File S2.pdf]

Supplementary File S2
Searching Strategy

1. Randomized Controlled Trial.pt.
2. Controlled Clinical Trial.pt.
3. (randomized or randomised).ab,ti.
4. placebo.ab,ti.
5. drug therapy.fs.
6. randomly.ab,ti.
7. trial.ab,ti.
8. groups.ab,ti.
9. 1 or 2 or 3 or 4 or 5 or 6 or 7 or 8
10. exp animals/ not humans.sh.
11. 9 not 10
12. exp dry eye syndromes/
13. (dry adj2 eye*).tw.
14. (ocular adj2 dry*).tw.
15. exp tears/
16. tear*.tw.
17. exp xerophthalmia/
18. xerophthalmi*.tw.
19. exp vitamin A deficiency/
20. (vitamin A adj3 deficien*).tw.
21. (avitaminosis a or retinol deficien* or hypovitaminosis A).tw.
22. exp keratoconjunctivitis sicca/
23. (Keratoconjunctiv* or kerato conjunctivitis).tw.
24. exp Keratoconjunctivitis/
25. limit 24 to yr="1966 - 1985"
26. exp Sjogren's syndrome/
27. ((Sjogren* or Sjoegren*) adj2 (syndrom* or disease*)).tw.
28. sicca syndrom*.tw.
29. exp Stevens Johnson syndrome/
30. (Steven* and Johnson and (syndrom* or disease*)).tw.
31. exp Pemphigoid, Benign Mucous Membrane/
32. Benign Muco* Pemphigoid*.tw.
33. (Cicatricial adj2 Pemphigoid*).tw.
34. blepharoconjunctiviti$.tw.
35. exp meibomian glands/
36. (meibomian or tarsal).tw.
37. exp lacrimal apparatus diseases/
38. (lacrima* or epiphora).tw.
39. or/12-23,25-38
40. exp Acupuncture/
41. exp Acupuncture Therapy/
42. exp Medicine, Chinese Traditional/

43. exp Acupressure/
44. (acupunctur* or acupressur*).tw.
45. (meridian* or moxi*).tw.
46. (electrostimulat* or electroacupunctur*).tw.
47. (electro* adj1 (stimulat* or acupunctur*)).tw.
48. acupoint*.tw.
49. exp Bloodletting/
50. (erjian or taiyang or EX-HN6 or EX-HN5).tw.
51. body needl*.tw.
52. (bloodletting or blood-letting or pricking blood).tw.
53. (Ear-apex or temporal region).tw.
54. qi.tw.
55. ((chinese adj3 medicin*) or TCM).tw.
56. or/40-55
57. 39 and 56
58. 11 and 57
